# Dry Gangrene From Transient Vaso-Occlusion in the Setting of Right-Sided Brachial Plexus Paralysis

**DOI:** 10.7759/cureus.87623

**Published:** 2025-07-09

**Authors:** Victoria A Volpe, David S Kirwin, Travis C Frantz, Willis H Lyford

**Affiliations:** 1 School of Medicine, Uniformed Services University of the Health Sciences, Bethesda, USA; 2 Department of Dermatology, Naval Medical Center San Diego, San Diego, USA

**Keywords:** brachial plexus syndrome, degloving injury, dry gangrene, neuropathy, upper extremity ischemia, vaso-occlusion

## Abstract

An 81-year-old woman with chronic right-sided motor and sensory neuropathy, a result of radiation-induced brachial plexus syndrome, presented with an asymptomatic degloving injury of her distal right index finger. This was rapidly followed by the onset of dry gangrene in both the index and middle fingers. The patient reported no history of trauma or thermal injury, noting that her hand was normal before sleep and that she discovered the injury upon awakening. Examination revealed circumferential skin loss on the index finger and dusky discoloration with bullae on the middle finger. Despite these findings, the right hand was warm and well perfused.

An extensive diagnostic evaluation, including echocardiography, CT angiography, Doppler studies, infectious and autoimmune workups, and skin biopsies, effectively ruled out embolic, infectious, and vascular etiologies. Given the absence of vascular obstruction and the patient’s history of waking with her arm in hyperflexed positions, the cause was determined to be pressure-induced transient ischemia.

This case represents the first documented instance of pressure-induced dry gangrene in the context of brachial plexus syndrome of the upper extremity since 1873. It underscores the critical importance of considering pressure-induced ischemia in patients with profound neuropathy, where a lack of protective sensation can lead to prolonged, unnoticed compression. Prompt recognition of such cases can prevent unnecessary interventions and guide appropriate management.

## Introduction

Gangrene is a medical condition characterized by necrosis of body tissue, typically resulting from ischemia or infection. The three most prominent types are dry gangrene, wet gangrene, and gas gangrene [1]. Dry gangrene is distinguished by tissue dehydration and discoloration, often presenting with a blackened appearance. This form results from compromised blood supply, most commonly due to peripheral artery disease in the lower extremities and embolism in the upper extremities [1,2]. Early intervention for dry gangrene is crucial to arrest further tissue necrosis and prevent progression to systemic toxicity. Management strategies include surgical intervention, antibiotic therapy, and supportive care.

This report details a unique case of an 81-year-old woman who developed dry gangrene in her right second and third distal fingers. The gangrene was caused by pressure-induced transient ischemia, occurring in the context of total sensory and motor neuropathy secondary to brachial plexus syndrome. Brachial plexus syndrome refers to a group of disorders caused by injury or dysfunction of the brachial plexus, a group of nerves originating from the spinal cord that control motor and sensory function of the upper extremity. It can result in profound sensory loss and muscle paralysis, which impairs a patient's ability to perceive and adjust to prolonged pressure, thereby increasing the risk of pressure-induced ischemia. To our knowledge, this is the first documented case of pressure-induced dry gangrene in the setting of upper extremity brachial plexus syndrome since a case report in 1873 [3].

## Case presentation

An 81-year-old woman with a past medical history significant for breast cancer, status-post definitive management with radiation therapy complicated by a radiation-induced brachial plexus syndrome, presented to the emergency department with an acute degloving injury to her right index finger. Due to chronic sensory and motor neuropathy of her right arm from the brachial plexus syndrome, the patient experienced no symptoms from the degloving injury. The patient was unaware of the etiology of the injury, stating that her finger was normal when she went to bed the previous night and that she discovered the skin loss upon awakening.

**Figure 1:**
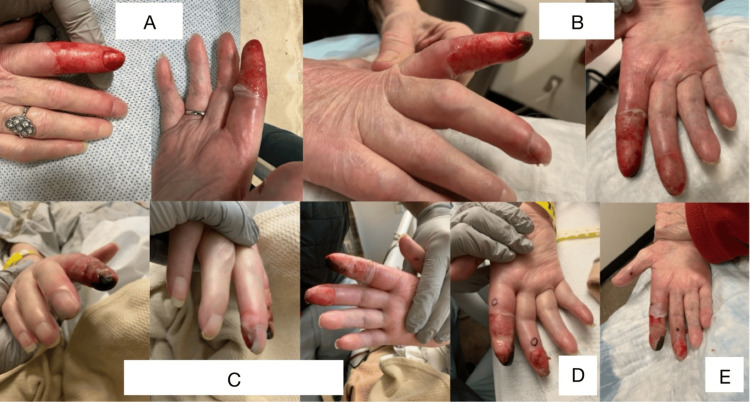
A) Emergency Department presentation. The patient exhibited circumferential skin loss on the distal right index finger, extending approximately 3 cm from the tip, along with a 1 cm bulla and dusky hyperpigmentation on the distal right middle finger. B) Twelve hours later, during follow-up in the dermatology clinic, dry gangrene of the distal right index finger was noted, with an increasingly dusky appearance of the distal right middle finger. C) Two days after presentation, on the patient’s first morning in the hospital, the dry gangrene and ischemia of the right distal index and middle fingers continued to worsen. D) Two punch biopsies were performed as indicated. E) At a one-week follow-up, the dry gangrene on the patient’s distal right second and third fingers had not progressed since discharge. The biopsy sites were healing well without complications.

Upon initial evaluation, she exhibited circumferential skin loss on the distal right index finger, extending approximately 3 cm from the tip with a sharp cutoff. Additionally, there was a 1 cm bulla and dusky hyperpigmentation on the distal right middle finger (Figure 1, section A). She denied any history of trauma or scald injury to her hand or digits. Her right arm and hand were warm and well perfused. Given her history of brachial plexopathy, she reported a predisposition to frequent right arm injuries and occasionally waking with her wrist in a kinked, hyperflexed position.

Twelve hours later, at close follow-up, the patient presented with a precipitously worsening dusky appearance of the middle finger, progressing to dry gangrene, which necessitated hospital admission for an expedited workup (Figure 1, section B). On her first day of hospital admission, the dry gangrene and ischemia of her right distal index and middle fingers continued to worsen (Figure 1, section C). Considering her history of aortic valve replacement and the ischemic presentation, an embolic event was initially suspected.

The patient underwent a comprehensive evaluation by a team of specialists, including those from cardiology, infectious disease, and vascular surgery. A transthoracic echocardiogram and CT angiography of the right arm were performed, neither of which showed evidence of embolism or vascular obstruction. Her vital signs were stable, and laboratory tests were negative for infection or systemic inflammation. Vascular surgery specialists performed Doppler imaging, which revealed normal blood flow in the right hand and fingers. With vascular occlusion and infectious etiologies largely excluded, punch biopsies were obtained to further evaluate for potential rheumatologic or embolic causes. Two punch biopsies of the right second and third fingers showed dermal fibrosis without vascular pathology or signs of infection (Figure 1, section D).

After thorough evaluation, common causes of distal finger necrosis, such as embolism, infection, vascular disease, drug-induced etiologies, and systemic autoimmune disease, were ruled out. Given the patient's history of total sensory and motor neuropathy in her right arm, supported by a neurological exam notable for flaccidity and complete loss of sensation, and her endorsement of finding her right hand in hyperflexed positions upon awakening, pressure-induced transient ischemia was determined to be the cause of the dry gangrene in her distal right index and middle fingers. After stabilization, she was discharged with close dermatology follow-up. One week later, the dry gangrene of her right second and third fingers remained stable and was in the process of healing (Figure 1, section E).

## Discussion

This case is notable as the first documented instance of pressure-induced dry gangrene in the setting of brachial plexus syndrome of the upper extremity since a similar case was reported in 1873 [3]. Dry gangrene typically results from compromised blood flow, often associated with conditions like atherosclerosis or thrombosis, leading to tissue dehydration and necrosis [1]. Unlike wet gangrene, which involves microbial invasion and decaying tissue, dry gangrene presents without infection [1]. Diagnosis relies on clinical evaluation, imaging, and tissue biopsy.

Upper extremity ischemia, though less common than in the lower extremities due to richer collateral circulation and lower atherosclerotic burden, presents unique diagnostic challenges [2,4-8]. Its causes can include arterial embolism, thrombosis, vasculitis, trauma, or iatrogenic factors. In the upper extremities, acute ischemia is frequently caused by embolic events, a contrast to the lower extremities, where thrombosis from atherosclerosis is more prevalent [2,5]. Diagnosis typically involves imaging studies such as Doppler ultrasonography, CT angiography, or conventional angiography to understand the underlying pathology.

The treatment of gangrene primarily involves arresting further tissue necrosis, which can be achieved by removing the inciting cause, restoring blood supply, and, if necessary, surgical debridement. In patients with motor and sensory neuropathy, prevention of pressure-induced ischemia includes frequent repositioning, use of pressure-relieving devices, meticulous skin care, and limb offloading to avoid unrecognized prolonged compression. Management of upper extremity ischemia may include anticoagulant therapy, endovascular interventions, and surgical revascularization to preserve limb function and prevent complications [4-8]. In this patient’s case, none of these interventions were indicated because there was no evidence of ongoing vaso-occlusion upon presentation. 

## Conclusions

This case presents an unusual scenario of dry gangrene in the distal right fingers, resulting from pressure-induced ischemia in the setting of total sensory and motor neuropathy due to right-sided brachial plexus syndrome. Common causes of ischemia, such as embolism, infection, vascular disease, and systemic autoimmune/autoinflammatory diseases, were ruled out through extensive diagnostic testing. This case highlights the importance of considering pressure-induced ischemia in patients with chronic neuropathy and suggests a need for careful evaluation of ischemic events in upper extremities, where the causes may differ from those typically observed in the lower extremities.
